# The conditional defector strategies can violate the most crucial supporting mechanisms of cooperation

**DOI:** 10.1038/s41598-022-18797-2

**Published:** 2022-09-07

**Authors:** Ahmed M. Ibrahim

**Affiliations:** Mansoura, Dakahlia Egypt

**Keywords:** Biotechnology, Computational biology and bioinformatics, Ecology, Evolution, Systems biology

## Abstract

Cooperation is essential for all domains of life. Yet, ironically, it is intrinsically vulnerable to exploitation by cheats. Hence, an explanatory necessity spurs many evolutionary biologists to search for mechanisms that could support cooperation. In general, cooperation can emerge and be maintained when cooperators are sufficiently interacting with themselves. This communication provides a kind of assortment and reciprocity. The most crucial and common mechanisms to achieve that task are kin selection, spatial structure, and enforcement (punishment). Here, we used agent-based simulation models to investigate these pivotal mechanisms against conditional defector strategies. We concluded that the latter could easily violate the former and take over the population. This surprising outcome may urge us to rethink the evolution of cooperation, as it illustrates that maintaining cooperation may be more difficult than previously thought. Moreover, empirical applications may support these theoretical findings, such as invading the cooperator population of pathogens by genetically engineered conditional defectors, which could be a potential therapy for many incurable diseases.

## Introduction

A long-standing puzzle in evolution theory is how cooperative behavior can evolve and persist within the selfish natural world. Once cooperation exists, it is always prone to exploitation by defective free riders who adopt selfish strategies for reaping the highest possible profit without paying the share. Thus, they invest most of their energy in reproduction. Therefore, cheaters could outcompete the cooperators and take over the population. Haldane pointed out that there was no general principle to solve this problem^1^. Since then, many partial mechanisms have been suggested, such as kin selection, group selection, reciprocity, policing, spatial structure, sanction, reward, and punishment^2–4^. Darwin himself suggested some core concepts of these mechanisms. This paper introduced conditional defector strategies that violate the kin selection, punishment, and spatial structure mechanisms.

### What are conditional defector strategies?

From a conceptual viewpoint, a conditional defector strategy may be any cheating strategy that could somehow cooperate. In other words, it is a cheater who pays additional costs or wastes a portion of the profit to survive. Therefore, it is not necessarily defective in all behaviors or at all times. However, it may cooperate in some behaviors now and then. Consequently, they are not pure defectors.

### Some forms of conditional defector strategies

#### Cooperate for the spread

Dispersal is beneficial because it decreases Kin competition, sustains resources, and outsets colonization. Without dispersal, the fate of all populations is extinction^5^. Hence, an intermediate dispersal rate of cooperators is essential for cooperation maintenance^6,7^. Furthermore, migration can induce an explosive outbreak of cooperation, even in a world of selfish individuals with various sources of randomness, starting with defectors only^8^. Recently^9^ showed for the first time that strategy-neutral migration triggered strategy oscillation in a two-strategy game even without the bridging of any transition strategies (states), thus defining a novel oscillating behavior fundamentally different from the conventional cyclic dominance previously found in a game of at least three or more strategies. Reference^10^ studied the impact of varying migration preferences in promoting cooperation. They revealed the role of orientation-driven migration, where individuals prefer to move closer to their neighboring cooperators or favor escape away from their neighboring defectors.

Nevertheless, dispersal is a costly behavior that increases the mortality of dispersers or decreases their fecundity^11,12^. Therefore, some studies have focused on the joint evolution of dispersal and cooperation^13^. Alternatively, there is a correlation between cooperation, dispersal rate, and dispersal cost^14^. Their findings asserted that the low dispersal cost selects against cooperation. Thus, if cheaters reduce their dispersal costs, they may turn the game against cooperators.

Usually, cheats are not good migrators because dispersal itself is a cooperative behavior. The migrators leave their suitable habitats to other unknown environments and face dangerous predators to colonize a new patch. Therefore, such behavior is costly for migrators. Nevertheless, its benefits are also gained by non-migrators because it decreases kin competition. Thus, dispersal is considered a cooperative behavior that naturally does not expect to be abundant in cheats. Hence, cheaters go extinct rapidly with the depletion of local patches they dominated without global prevalence like cooperators; this might be the fundamental problem of cheaters. However, the probable solution is adopting a conditional defection strategy wherein free riders would cooperate only for the spread. The actors of this selfish strategy would have a high dispersal rate with the lowest possible cost because they share migration costs. Thus, the exploitation rate of public goods and interactions among defectors and cooperators will increase. In other words, the conditional defectors can exclusively cooperate in all collective behaviors related to migration (coalition dispersal) but defect otherwise. Therefore, these selfish successful migrators can convert the structured meta-population into a well-mixed game and violate the spatial structure mechanism.

In fact^15^, is considered empirical evidence to assume that individuals reduce their dispersal costs by sharing it. Thereby, they can achieve successful migrations. Additionally, in metastatic cancer, migrating in groups (coalition dispersal) raises the efficacy to 50-fold more than individual dispersal^16,17^.

#### Pay for the escape

Conditional defectors can pay some of their wealth or waste some profits to escape punishment by producing substances to mislead punishers. Or possession of the significant tag that marks cooperators. Similarly, by reducing their payoff to be more similar and familiar to cooperators. If cheaters reduced the benefits, it might be hard to have been noticed by a quorum-sensing system or other defense mechanisms. It is considered a kind of imitation or tag-based decision that prevents cooperators from detecting and punishing defectors.

Those cheaters pay a cost to escape sanctions or reduce the accuracy of the monitoring/punishment system. Therefore, they can merge with cooperator populations accordingly, violating punishment and kin-selection mechanisms.

## Methods

We used two agent-based simulation models to investigate the concepts of "cooperate for the spread" and "pay for the escape," both were *net logo* models created by Dr. Susan Hanisch.

Afterward, we modified the first model to represent the concept of sharing the dispersal costs. We used the second model without modifications. Instead, we assigned definite values of some parameters that highlight the pay for the escape strategy.

### First model

The original model was entitled "Evolution and patchy resource"^18^. She first developed it for educational purposes. It illustrates the concepts of cooperator-cheater competition, natural selection, spatial structure mechanisms, multilevel selection, and founder effects.

#### Changeable variables

- Distance-resource-areas: the distance between the centers of the resource areas.
- Size-resource areas: the size of resource areas as a radius in the number of patches.
- Living costs: the costs that each agent has to deduct from energy per iteration for basic survival.
- Mutation rate: The probability that offspring agents have different traits than their parents.
- Evolution: the ability of agents to produce offspring.

#### Constant variables

- The number of patches is 112 × 112 patches.
- Carrying capacity per patch: Resource = 10, Agents = 1
- The growth rate of the resource = 0.2
- The resources on a patch regrow by a logistic growth function up to the carrying capacity: New resource level = current resource level + (Growth-Rate × current resource level) × (1 - (Current resource level/carrying capacity)).
- The cost for producing offspring is ten subtracted units of energy.
- The initial level of energy of agents is set at living costs.

#### Role of randomness

- Agents are distributed randomly in resource areas at the beginning of a simulation.
- Sustainable behavior is distributed randomly with a probability of percent sustainables among the initial agent population.
- The order in which agents move and harvest within one iteration is random.
- Agents move to a randomly selected patch if several patches fulfill the objectives.
- The order in which agents produce offspring within one iteration is random.
- Agents reproduce offspring with a probability of (0.0005 × Energy).
- Agents place offspring on a randomly selected unoccupied neighboring patch.
- Offspring mutate with a potential mutation rate.

#### Model processes

In each iteration, each agent moves around in random order. There are three likelihoods:

- If there are no unoccupied patches in a two-patch radius, they stay on the current patch.
- If there are unoccupied patches with resources amounting to more than living costs, the agents move to them.
- If the resource amount is less than the living costs, the agents move randomly to other unoccupied patches.

The agents harvest the resources from separated patches to gain energy for metabolism and proliferation. If the energy level of any agent falls to zero, it dies. The cooperator type harvests half of the resource, while the greedy type consumes 99%.

The living costs are deducted from the energy amount of the agent constantly everywhere all the time. This process occurs whether an agent moves within the patch, between the patches, or even not. Therefore, the model does not consider dispersal cost explicitly.

If there is an unoccupied neighbor patch, the agent can reproduce with a probability of 0.0005 of his energy, place the offspring on the unoccupied neighbor patch, and then transfer ten units of the energy to his offspring.

Resources regrow only on resource patches. When the resource amount is more than or equal to 0.1, then it regrows. When the resource is less than 0.1, its value is set to 0.1.

#### Output diagrams and monitors

- The average energy of agents: average energy levels of sustainable and greedy agents, resulting from resource harvest minus living costs and reproduction.
- Trait frequencies: the relative frequencies of sustainable and greedy agents in the total population, resulting from mutations, different reproduction rates, and death.
- Agent population: the absolute number of the total population size resulting from reproduction and death.

#### Modifications

In the first modification, we added a different type of cost that agents only incur when they disperse from one patch to another (in-between the patches). It is the slider entitled "dispersal costs".

In the second modification, we added another sharing dispersal costs tool to reduce them by dividing their value by the number of included agents (flock-mates) in the identified range from the same type. It is the slider entitled "group-dispersal-range." which is the flock mate's areas as a radius in the number of patches. Therefore, changing the value of the group dispersal range will change the area around every agent. Accordingly, the number of its flock mates who share the dispersal costs also adjusts.

The group dispersal range is not confined to greedy agents but applies to all agents. Therefore, it represents the case of the wild-type cooperators who can also cooperate for the spread. The group dispersal range also does not only target the agents in between patches. However, it counts the agents inside and outside the patches. For example, once an agent starts its dispersion with a determined range containing ten agents, four from another type, three non-dispersal agents from the same type that existed inside a patch, and three dispersal agents from the same type outside the patches. The dispersal costs for this agent will be divided by 6.

Our assumption that non-dispersal agents at the pre-departure stage share dispersion costs with dispersal agents; seems justified because they reap mutual benefits by reducing kin competition inside patches if they promote the migrators. However, can agents remotely pay the dispersion costs? Yes. For instance, some bacterial species can trigger the migration of other species if located in their vicinity, even if the two bacterial colonies are separated by a barrier^19,20^ or if they are non-motile^21^. On the other hand, dispersion is an extended process with many factors, including escape from predators, suppression of host defense mechanisms, and production of biosurfactants to reduce surface tension to facilitate motility. Therefore, the agent's contribution (inside/outside the patches) to support such factors is considered a shared dispersal cost.

Finally, cheaters can arise within cooperator patches by mutation or immigration. Therefore, to investigate the efficacy of migration, the mutation rate value should be 0 to cancel its effect in the meta-population dynamics.

### Second model

The model is entitled "Evolution, resources, monitoring, and punishment."^22^ is a simulation of a population with four types of agents competing for the same resource. It demonstrates many concepts, such as kin selection, cooperation, selfishness, public good, monitoring, punishment, sharing the costs, positive/negative frequency-dependent selection, and multilevel selection. The four agent colors and types: (1) Red: greedy, non-punishing. (2) Orange: greedy, punishing. (3) Turquoise: sustainable, non-punishing. (4) Green: sustainable, punishing.

Punishing agents can perceive other agents in their environment to some degree (perception accuracy) and react to their behavior. There are three kinds of punishment: Punishers can kill agents with greedy harvesting behavior, stop them from harvesting in the next iteration, or have them pay a penalty fee to their neighbors.

Agents have a cost (energy) to pay for, both detection and punishment, so this behavior is altruistic. Punisher agents of one type share punishment costs equally.

#### Changeable variables

- Death rate: the probability that agents die independent of their energy level.
- Carrying capacity: the maximum amount of resource units on a patch from 1 to 100.
- Growth rate: the rate at which resources on patches regrow. The maximum sustainable yield is calculated based on the carrying capacity and growth rate.
- Harvest-sustainable: the number of resource units harvested by sustainable agents.
- Harvest-greedy: the number of resource units harvested by sustainable agents.
- Perception accuracy: the probability with which punishing agents notice greedy agents.
- Costs-perception: the costs in units of energy, punishing agents have to pay for perceiving other agents.
- Costs-punishment: the costs as units of energy that punishing agents have to pay in each iteration to punish other agents. All punishing agents of an agent divide the costs of punishment.
- Punishment: the kinds of punishing behavior that punishing agents perform.
- Fine: if the kind of punishment is "pay fine", the fine in energy units that punished agents have to pay (shared between all their neighbors).
- Living costs and mutation rate: see the first model.

#### Constant variables

- The number of patches: There are 60 × 60 patches in the world.
- The initial energy level of agents is set at living costs + 1.
- The initial number of resource units on a patch is set to the carrying capacity.
- The resources on a patch regrow: see the first model.

#### Role of randomness

* In addition to items in the first model.

- Agents take on their traits (harvest preference and ability to notice and punish) randomly based on the probability of percent-sustainable and percent-punishers.
- The order in which punishing agents notice greedy agents within one iteration is random.
- Greedy agents are noticed by punishing agents with a probability of perception accuracy.
- The order in which detected greedy agents are punished within one iteration is random.
- Agents produce offspring with a probability of (0.001 × Energy).
- Agents die with a probability of (death-rate).

#### Model processes

In each iteration, each agent attempts to harvest resources from the patches it is on and the eight neighboring patches until the harvest preference level is reached, except for the punished agent with the sanction (suspend harvest once), its harvest amount = 0 in the current iteration. If the amount of resources available is lower than the amount that the unpunished agent attempts to harvest. Then, the agent moves to a neighboring unoccupied patch with the most resources after losing one energy unit as a move cost.

Punishers pay the costs of perceiving the greedy agents. The greedy neighbors have been noticed with the probability of perception accuracy. The agent lost an amount of energy as living costs. The agent dies with the likelihood of death rate or if the energy level falls to zero.

If there is an unoccupied neighbor patch, the agent can reproduce with a probability of 0.001 of its energy, place the offspring on the unoccupied neighbor patch, and then transfer half of its energy to its offspring that mutate according to the probability of the mutation rate.

Resources regrow on all patches. When the resource amount is more than or equal to 0.1, then it regrows. When the resource is less than 0.1, its value is set to 0.1.

#### Output diagrams and monitors

- Populations (% of carrying capacity): the state of the resource and the agent population in the world as a percentage of total carrying capacity resulting from resource harvesting behavior and resource regrowth, agent reproduction, and death.
- Average harvest per iteration: the average harvested amounts of agents per iteration by trait, resulting from harvested resource units, minus costs for monitoring and punishing (for punishing agents), minus fines (for punished agents in case of punishment “Pay fine”)
- The average energy of agents and trait frequencies: see the first model.

#### How does the model represent a conditional defector strategy?

The model aims to highlight the role of kin selection and punishment mechanisms in supporting cooperation evolution against cheats. We did not need to modify the model but just thought about what the conditional defector should do to upside down the game. The answer was to pay for the escape.

For instance, if the standard Harvest-greedy of a cheater (greedy, non-punishing) was 13 and the Perception-accuracy of its actual punishers was 75%. Now suppose this cheater faces troubles, and it cannot dominate. However, if it gives up some of its profit to become 12, to escape punishment, and to reduce the perception accuracy to 60%, it could dominate and take over the population.

The conditional cheater can pay something and reduce its profit to escape punishment by reducing perception accuracy if there is a positive correlation between these two variables. Therefore, this model is appropriate if it can support/deny such a correlation.

## Results

All experiments were carried out under the *Net Logo* Behavior Space. All data analyses were carried out via a *Python* library called *Glueviz* and *Excel*.

### The experiments of the first model

The default values of the variables: mutation rate = 0 (to investigate only the effect of dispersal). Dispersal costs = 8 (high value). The agent's shape is Bacteria. Size-Resource-Areas = 4 (Relatively small). Living costs = 1. Percent-Sustainables = 90% (most of the population initially consists of cooperators). Number-Agents = 80 (started number). Distance-Resource-Areas = 20, (Relatively far). The evolution switch is true (natural selection is working). Group dispersal range = 0, 30, 50, 70, 100, 150, and 200.

Seven experiments were carried out with 63 runs. Fifteen repeated runs for group dispersal range = 0, and 8 repeated runs for each other value. Approximately all runs with group dispersal range = 0 finished in favor of cooperators and the extinction of cheaters; as expected, cheaters cannot sustain their patches and cannot arrange successful migrations to other patches due to the high dispersal costs.

**Figure 1:**
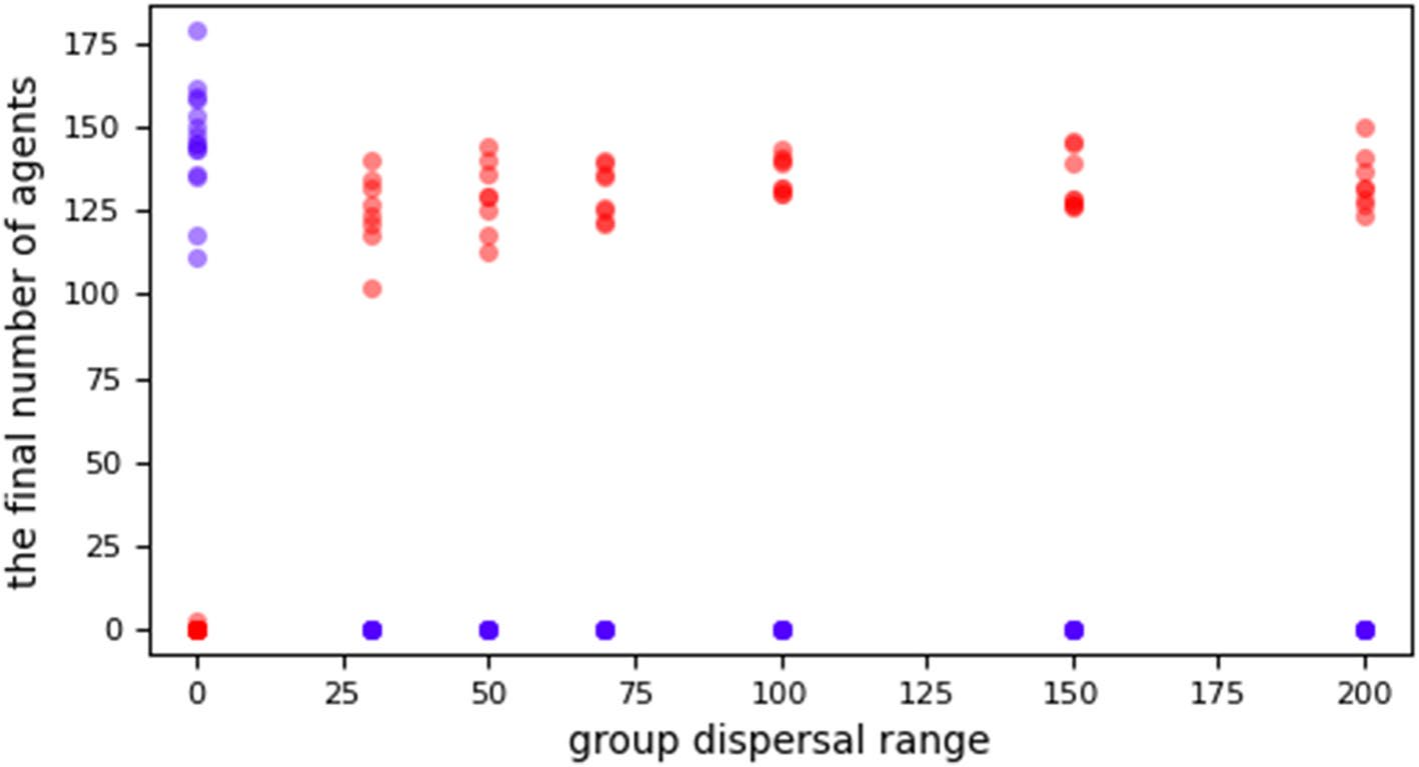
The final numbers of cheaters (red dots) and cooperators (blue dots) at different group dispersal ranges: cheaters could thrive only when they started to share the dispersal costs to some degree. However, when the group dispersal range = 0, each cheater pays the dispersal costs by itself. Therefore, cheaters cannot arrange successful migrations and cannot violate the spatial structure mechanism. Hence, they encounter local extinction at their patches.

**Figure 2:**
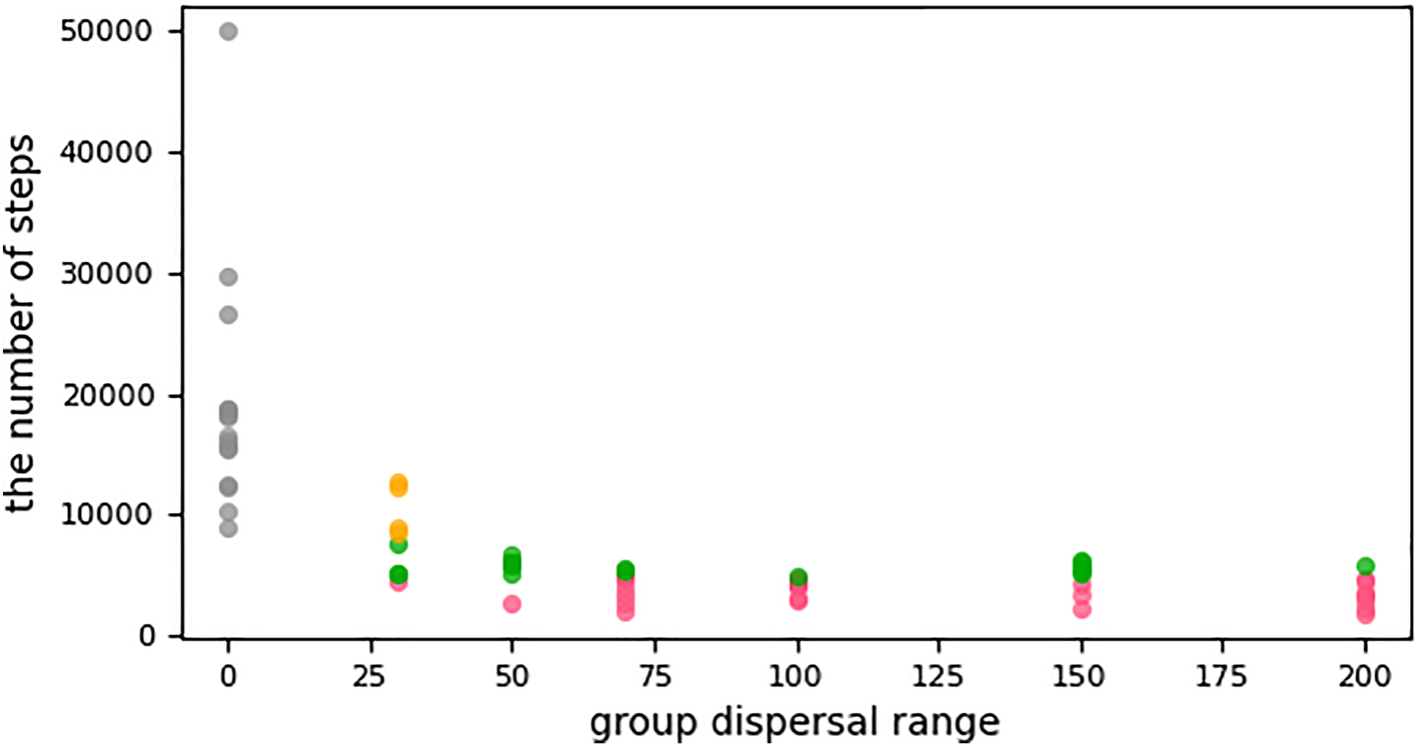
The runs that finished in favor of cooperators: (gray dots) most of these runs finished from 9000 to 30,000 steps by the complete extinction of cheaters, except one run reached the stop limit of our experiments at 50,000 steps, as three cheater agents succeeded to persist. The group dispersal range was 0 in all these runs. Therefore, cheaters cannot violate the spatial structure mechanism. The runs that finished in favor of cheaters by the complete extinction of cooperators: (1) (orange dots), runs finished after 8220 steps. (2) (green dots), finished from 5000 to 8220 steps. (3) (pink dots), finished before 5000 steps.

**Figure 3:**
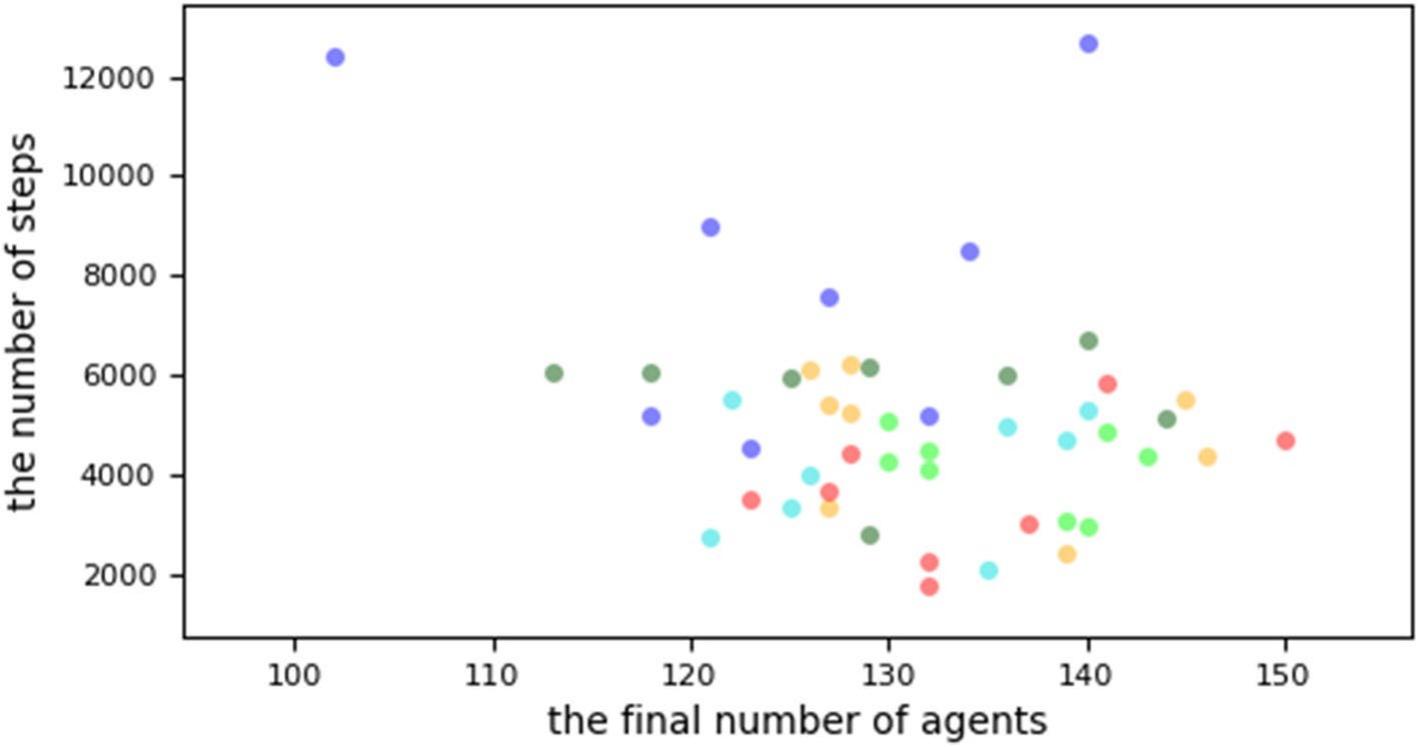
Different group dispersal ranges: (blue dots), group dispersal range = 30. (dark green dots), group dispersal range = 50. (sky blue dots), group dispersal range = 70. (light green dots), group dispersal range = 100. (orange dots), group dispersal range = 150. (red dots), group dispersal range = 200. Cheaters outcompeted cooperators in all of these runs. However, the extinction of cooperators is likely to be done more quickly, with fewer steps in the higher group dispersal ranges.

This situation significantly changed in the rest of the runs of group dispersal, ranging from 30 to 200, where cheaters could share the dispersal costs. Consequently, all these runs ended in favor of cheaters, and all cooperators were extinct. Figure 1. Additionally, cheaters in these runs outcompete cooperators quickly with fewer steps, as long as the group dispersal range increases from 30 to 70. Then, the average number of steps is somewhat convergent for the group dispersal range from 70 to 200. Figure 2. In addition, Fig. 3.

The results follow the intuitive predictions that cheaters could thrive, violate the spatial structure mechanism, and dominate the whole meta-population as long as they could cooperate to decrease the dispersal costs.

### The experiments of the second model.

The default values of the variables: mutation rate = 1%. The kinds of Punishment are "suspended harvest once", "pay fine", or "kill". The fine if existed = 5. Carrying capacity = 100. Number Agents = 250, (started number). Costs perception = 0.5. Growth rate = 0.3. Costs-punishment = 0.8 Percent-Punishers = 20%, (started ratio). Harvest-sustainable = 7. Percent-Sustainables = 99% (most of the population initially consists of cooperators). Living costs = 4. Death rate = 1. Perception-accuracy%. Harvest-greedy  (see Table 1).

**Figure 4:**
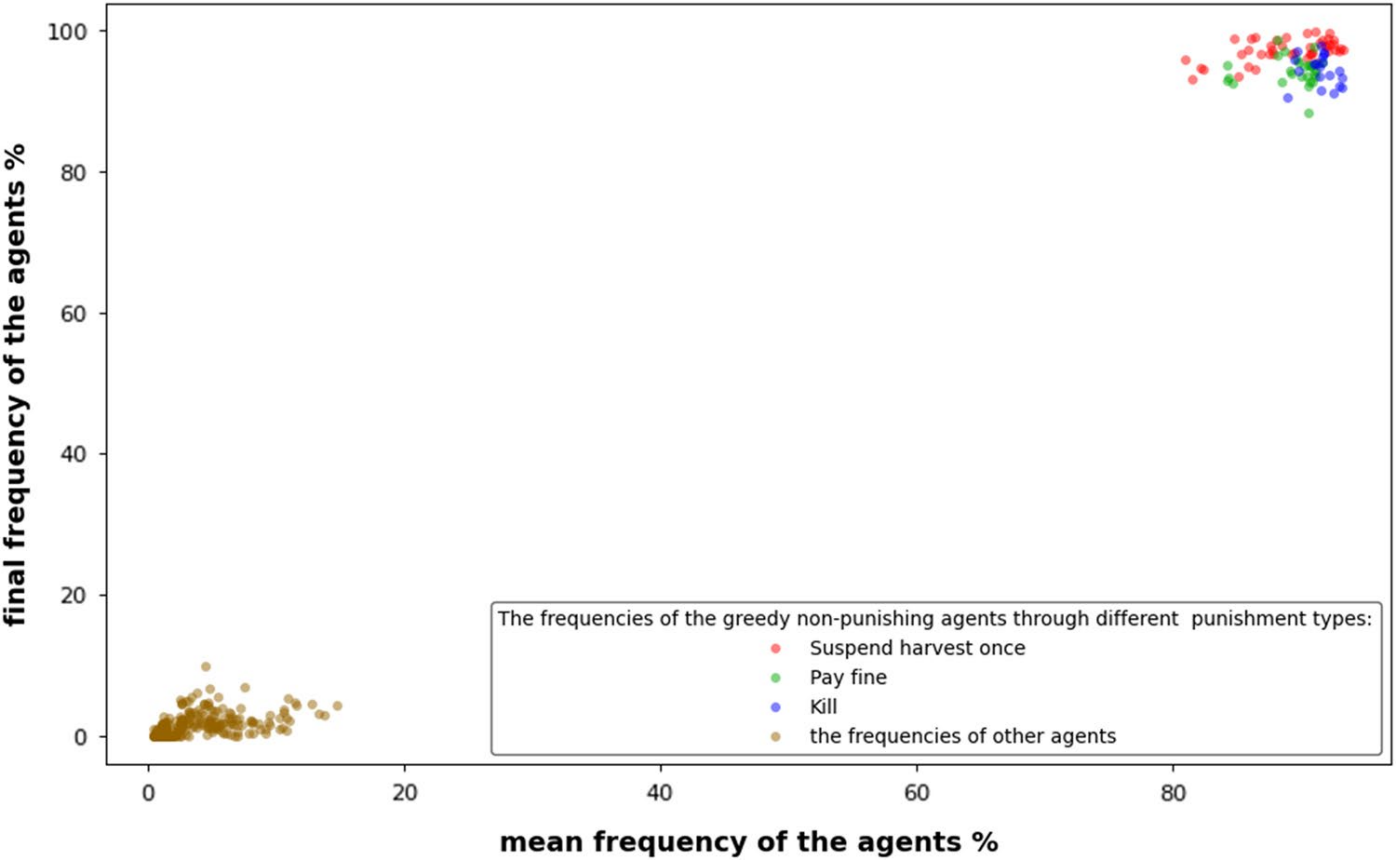
In the upper right, the frequencies of the greedy non-punishing agents through different experiments on three types of punishment: (1) Suspend harvest once (red dots). (2) Pay fine (green dots). (3) Kill (blue dots). The final frequencies of greedy non-punishing agents were above 90% in most runs (except one run for pay fine type was 88%). The mean frequencies of greedy non-punishing agents were above 80% for all runs. In the lower left, the frequencies of the other agents (brown dots): (1) Sustainable, punishing. (2) Sustainable, non-punishing. (3) Greedy, punishing.

The runs were 15,000 ticks (iterations or steps) for the punishment types (suspended harvest once and pay fine). However, there were 30,000 ticks for the third type (Kill). The experiments began with a 99% frequency of cooperators, ending with greedy, non-punishing agents taking over the population. The final frequency of greedy non-punishing was above 90% in most runs, and the mean frequency of all steps was above 80% (Fig. 4). We excluded 100% accuracy, as it seems to us that there is no such perfect monitoring case in nature. In the first type of punishment (suspended harvest once), we began with 99% accuracy and then degraded to reach 30%, parallel to similar degradation in the greedy harvest amount from 15 to 9, Table 1. In the second type of punishment (pay fine), we used the same values for greedy harvest amount and perception accuracy as the first type of punishment. The results of the two types were similar when the fines were five or less. In the third type of punishment (kill), we used different values for perception accuracy (from 70 to 30%) and greedy harvest amount (from 40 to 12) Table 1.

**Table 1:** Experimental details of the second model.

Number of steps	Fine	Punishment type	Harvest-greedy	Perception-accuracy%	Number of runs	Experiment
15,000	0	Suspend harvest once	15	99	8	1
15,000	0	Suspend harvest once	14	90	8	2
15,000	0	Suspend harvest once	13	70	4	3
15,000	0	Suspend harvest once	12	60	4	4
15,000	0	Suspend harvest once	11	50	8	5
15,000	0	Suspend harvest once	10	40	8	6
15,000	0	Suspend harvest once	9	30	4	7
15,000	5	Pay fine	15	99	4	1
15,000	5	Pay fine	14	90	4	2
15,000	5	Pay fine	13	70	4	3
15,000	5	Pay fine	12	60	4	4
15,000	5	Pay fine	11	50	4	5
15,000	5	Pay fine	10	40	4	6
15,000	5	Pay fine	9	30	4	7
30,000	0	Kill	40	70	4	1
30,000	0	Kill	34	65	4	2
30,000	0	Kill	30	59	4	3
30,000	0	Kill	16	49	4	4
30,000	0	Kill	12	30	4	5

The correlation coefficient (r) = 0.99 (rounded to 2 decimal places) for the first and second punishment types (suspend harvest once/pay fine). In addition, (r) = 0.95 (rounded to 2 decimal places) for third punishment type kill.

**Figure 5:**
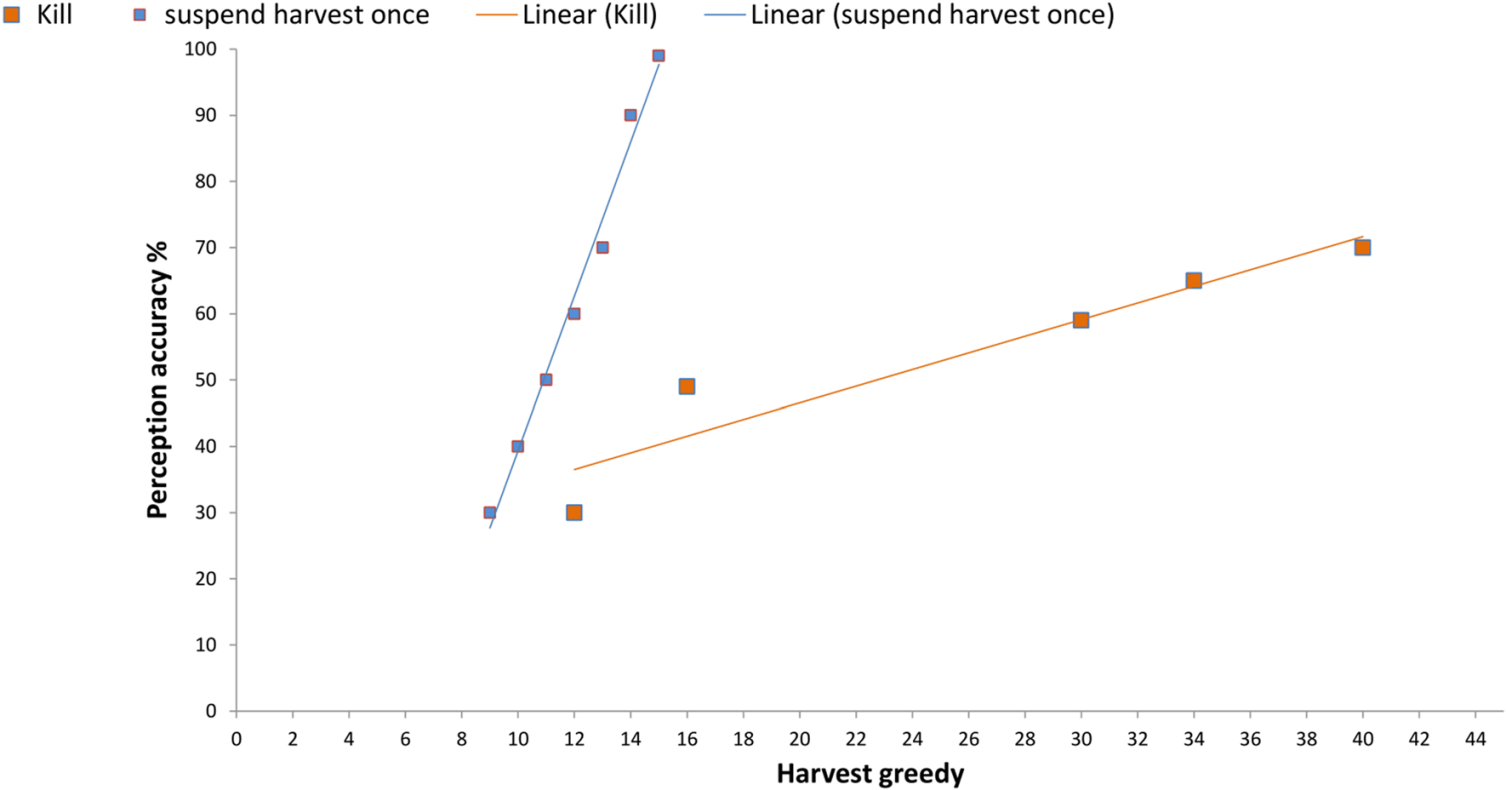
A strong positive correlation between the variables (harvest greed and perception accuracy) through different punishment types: (1) The first type, "suspend harvest once" (small blue squares). The second type, "pay fine", is the same. (2) The third type "kill" (large orange squares). (3) The blue line is the linear relationship of the selected values through the first/second type of punishment. (4) The orange line is the linear relationship of the selected values through the third type of punishment.

Our findings demonstrate a strong positive correlation between the two variables (harvest greed and perception accuracy). The correlation coefficient (r) = 0.99 for the first and second punishment types. (r) = 0.95 for the third punishment type (Fig. 5, Table 1).

The selected correlated values led to the dominance of greedy non-punishing agents through the three types of punishment. The dominance of cheaters here means they can violate kin selection and punishment mechanisms when they pay to escape punishment.

## Discussion

The frequent dominance of conditional defection strategies in the computational experiments that we have conducted declares the failure of several well-established and crucial mechanisms responsible for augmenting cooperation, such as kin reciprocity, punishment, and spatiality. Therefore, it can be said that maintaining cooperation may be far harder than previously thought.

The two models^18,22^ we followed paved the way by encapsulating substantial problems of cooperation as well as the most crucial supporting mechanisms. These models aimed to assert the efficient role of kin selection, punishment, and spatial structure mechanisms for supporting cooperators against cheats. The novelty of the present article is that it takes the results to the reverse side by designing the conditional defection strategies and modifying the program codes of the previous models to involve the concept of sharing the dispersal costs. It also reveals the effective behavior of paying to escape different types of punishment.

The two forms we presented in this article are simple and general. However, the concept of the conditional defection strategy is much broader. For example, the zero-determinant (ZD) extortion strategy is also a conditional defector strategy if it has a tag-based decision to cooperate with relatives who adopt the same (ZD) extortion strategy but cheat otherwise. At that time, it could be stable and win the game against the opponent's strategies^23^. In addition, when selfish strategies can modulate benefits and costs, they can outcompete tit-for-tat and generous strategies^24^. On the other hand, cheaters who can increase their dispersal rate without decreasing the dispersal costs often cannot achieve triumph. Therefore, they cannot drive the cooperators (wild type) to go extinct or even harm themselves if the benefits of exploitation do not offset the costs of dispersal. For instance, the social parasite of *P. punctatus* ants is a wingless cheater queen. Although it has a high dispersal rate, it has costly migration on foot for long distances. Therefore, the colonies persisted for a long time instead of the supposed rapid collapse of the whole population^25^.

The findings of the present paper suggest a potentially therapeutic application. Conditional defectors can be used as suicidal agents to drive the population of pathogens into the self-destruction process. From an evolutionary perspective, tumors or microbes are considered populations of cooperating cells that struggle for survival by adopting many collective costly actions to produce the intrinsic common resources^26–28^. However, conditional defectors can violate the crucial mechanisms that support cooperation. Thereby, cheaters take over the population. Then they also go extinct after cooperators because they cannot do the necessary collective actions. Undoubtedly, the production of the common resources or the public good we meant is not independent of cooperators, as in the two models in the present paper. Instead, its production ought to rely on cooperators. For example, the essential excretions of microbes deplete after the extinction of the cooperator. Cheaters can drive the whole population to go extinct; it is a well-established evolutionary prediction. This robust outcome appears in many theoretical and empirical studies and is known as the tragedy of the commons or evolutionary suicide^29–31^. This phenomenon can occur if free riders have a fitness advantage over cooperators (wild-type) in an environment set by the cooperators. Creating evolutionary suicide within pathogen populations would mean the end of infections or even endemics, as cheaters are not static chemical substances but infectious transmissible organisms.

It is not the first time someone has suggested using cheaters in attacking pathogens as a cooperator population. For instance, Brown et al.^32^ suggested trojan horse therapy to reduce the virulence of pathogens or release beneficial medical substances inside its colonies.

Weinberger et al.^33^ suggested therapeutic interfering particles (TIPs) or hijacker therapy. It is a therapeutic utilization for defective interfering particles (DIPs) that are molecular parasites of viruses or incomplete RNA particles lacking essential packaging elements. It is believed to defeat HIV and other viruses (such as SARS-CoV-2). Moreover, DIPs are antivirals that can be transferred from one person to another until the end of endemicity in infected areas such as sub-Saharan Africa^34^.

Archetti^35^ suggested autologous therapy. It aims to increase the diffusion range of the growth factors that the tumor is excreting. Hence, this could increase the tumor's vulnerability to exploitation^36^.

Domingo-Calap et al.^37^ manipulated a defector strain of *vesicular stomatitis* virus called Δ51. It does not excrete a costly enzyme that suppresses interferon. So it could defeat the wild type. Then led to the tragedy of the commons.

Other treatments and descriptive game-theoretic models of cancer are reviewed here^38^.

To date, many previous papers have suggested closely related ideas. However, the defense mechanisms of cooperators are always a huge obstacle. We think now conditional defector strategies can surpass these obstacles.

## Data Availability

The datasets generated or analyzed during the current study include the three codes of Net Logo 6.1.1 (both codes of Dr. Susan and my own modified one) are available and accessible at the following *CoMSES* Computational Model Library: https://www.comses.net/codebases/8437728b-3e1f-46f3-80be-65f4f5909d81/releases/1.0.0/.
